# High-resolution and high-sensitivity X-ray ptychographic coherent diffraction imaging using the CITIUS detector

**DOI:** 10.1107/S1600577523004897

**Published:** 2023-08-01

**Authors:** Yukio Takahashi, Masaki Abe, Hideshi Uematsu, Shuntaro Takazawa, Yuhei Sasaki, Nozomu Ishiguro, Kyosuke Ozaki, Yoshiaki Honjo, Haruki Nishino, Kazuo Kobayashi, Toshiyuki Nishiyama Hiraki, Yasumasa Joti, Takaki Hatsui

**Affiliations:** a International Center for Synchrotron Radiation Innovation Smart (SRIS), Tohoku University, 2-1-1 Katahira, Aoba-ku, Sendai 980-8577, Japan; b Institute of Multidisciplinary Research for Advanced Materials (IMRAM), Tohoku University, 2-1-1 Katahira, Aoba-ku, Sendai 980-8577, Japan; c RIKEN SPring-8 Center, 1-1-1 Kouto, Sayo-cho, Sayo-gun, Hyogo 679-5148, Japan; d Department of Metallurgy, Materials Science and Materials Processing, Graduate School of Engineering, Tohoku University, 6-6-2 Aoba-yama, Aoba-ku, Sendai 980-8579, Japan; e Japan Synchrotron Radiation Research Institute, 1-1-1 Kouto, Sayo-cho, Sayo-gun, Hyogo 679-5198, Japan; RIKEN SPring-8 Center, Japan

**Keywords:** pychographic coherent diffraction imaging, CITIUS

## Abstract

Ptychographic coherent diffraction imaging (PCDI) is a synchrotron X-ray microscopy technique that provides high spatial resolution and a wide field of view. To improve the performance of PCDI, the performance of the synchrotron radiation source and imaging detector should be improved. In this study, ptychographic diffraction pattern measurements using the CITIUS high-speed X-ray image detector and the corresponding image reconstruction are reported. X-rays with an energy of 6.5 keV were focused by total reflection focusing mirrors, and a flux of ∼2.6 × 10^10^ photons s^−1^ was obtained at the sample plane. Diffraction intensity data were collected at up to ∼250 Mcounts s^−1^ pixel^−1^ without saturation of the detector. Measurements of tantalum test charts and silica particles and the reconstruction of phase images were performed. A resolution of ∼10 nm and a phase sensitivity of ∼0.01 rad were obtained. The CITIUS detector can be applied to the PCDI observation of various samples using low-emittance synchrotron radiation sources and to the stability evaluation of light sources.

## Abstract 2

High-resolution and high-sensitivity X-ray ptychographic coherent diffraction imaging is demonstrated using a CITIUS high-speed X-ray image detector.

## 1. Introduction

Coherent diffraction imaging (CDI) is a lensless microscopy technique that reconstructs a sample image by performing iterative phase retrieval calculations on a computer based on a two-dimensional diffraction pattern observed in the far field when the sample is illuminated with coherent X-rays (Chapman & Nugent, 2010 ▸; Miao *et al.*, 2015 ▸). Ptychographic CDI (PCDI) is a scanning-type CDI technique that can be used to observe a spatially extended sample. Therefore, it can be applied to various sample observations and probe characterization (Rodenburg *et al.*, 2007 ▸; Pfeiffer, 2018 ▸). Additionally, it has been applied in a wide range of research fields, including biology (Giewekemeyer *et al.*, 2010 ▸; Deng *et al.*, 2015 ▸), materials science (Shapiro *et al.*, 2014 ▸; Hirose *et al.*, 2019 ▸) and devices (Holler *et al.*, 2017 ▸), with a field of view on the 10 µm scale and a spatial resolution of approximately 10 nm. Further improvement of PCDI performance is challenging.

The key performance characteristics of PCDI, such as spatiotemporal resolution and sensitivity, are highly dependent on the performance metrics of the synchrotron radiation source used (*e.g.* coherent flux and stability) and the image detector (*e.g.* photon count rate, sensitivity and area). Since synchrotron radiation sources produce partially coherent light in space and time, slits for generating virtual light sources are necessary, in addition to monochromators. Additionally, focusing devices play a significant role in forming high-intensity coherent X-ray beams. Spatial resolution at a 10 nm level has been achieved by PCDI using focusing devices such as total reflection mirrors (Takahashi *et al.*, 2011 ▸), Fresnel zone plates (Vila-Comamala *et al.*, 2011 ▸) and refractive lenses (Schropp *et al.*, 2012 ▸) thus far. Recent technological advances in low-emittance storage rings have increased the coherent flux available at new synchrotron facilities (Johansson *et al.*, 2021 ▸) and upgrades to existing facilities (Pacchioni, 2019 ▸). Further performance improvements in PCDI are expected in the near future.

In contrast, with regard to image detectors, the advent of single-photon-counting pixel detectors such as EIGER (Dinapoli *et al.*, 2011 ▸) and Lambda (Pennicard *et al.*, 2012 ▸) has significantly improved the measurement throughput of PCDI (Guizar-Sicairos *et al.*, 2014 ▸; Wilke *et al.*, 2014 ▸). However, photon count rates have reached their limits of approximately 1 Mcounts s^−1^ pixel^−1^ because of pile-up challenges inherent in photon-counting types, which necessitates the use of attenuators (Wilke *et al.*, 2013 ▸; Reinhardt *et al.*, 2017 ▸). Integrating-type detectors can operate regardless of the instantaneous count rate limitation (Hatsui & Graafsma, 2015 ▸). Tate *et al.* (2013 ▸) reported the demonstration of a quasi-integrating-type detector MM-PAD by implementing a mixed-mode in-pixel circuitry. Additionally, high-flux PCDI using MM-PAD has been reported (Giewekemeyer *et al.*, 2014 ▸). One of the other development programs to realize count rate beyond the photon counting limit is CITIUS (Hatsui *et al.*, in preparation), which has native integrating-type pixels.

In this study, we first demonstrate high-spatial-resolution and high-sensitivity PCDI using the CITIUS detector at SPring-8. Furthermore, we conduct a quantitative evaluation of the spatial resolution and sensitivity of PCDI.

## 2. Experimental

**Figure 1:**
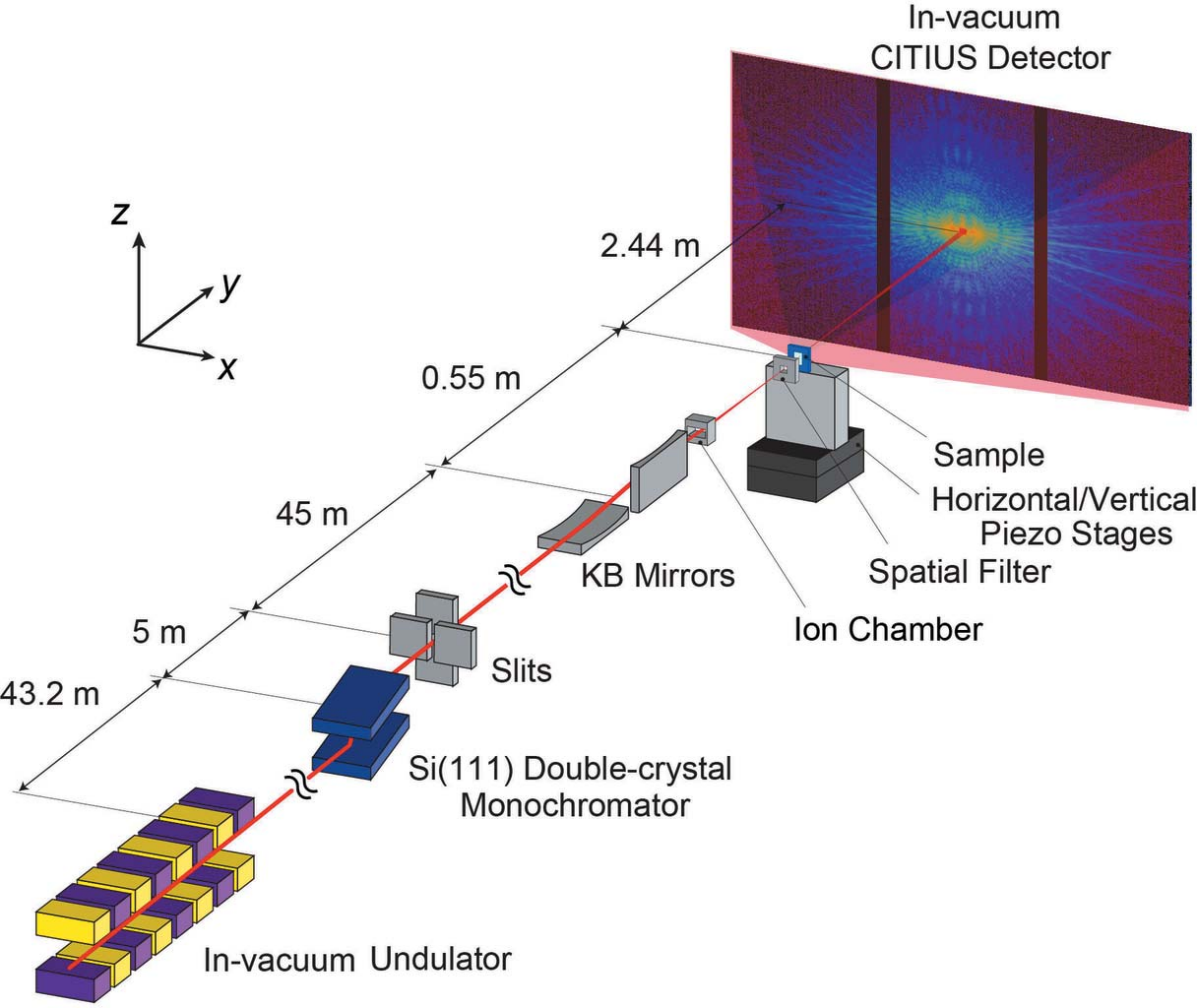
Experimental setup of the ptychographic measurement system with the CITIUS detector.

PCDI measurements were performed at the BL29XUL beamline (Tamasaku *et al.*, 2001 ▸) at SPring-8. Fig. 1 ▸ shows the experimental setup. Synchrotron radiation emitted from an in-vacuum undulator device was monochromated to 6.5 keV by a Si (111) double-crystal monochromator, and the X-ray beam was cut out with slits. The size of the light source in synchrotron radiation was determined by the size of the electron beam. The width of the Gaussian distribution defined the size, which was 301 µm in the vertical (V) direction and 6 µm in the horizontal (H) direction when all gaps of insertion devices were opened. Based on the van Cittert–Zernike theorem, the transverse coherence length at the slit position was approximately 17 µm (H) × 800 µm (V). The X-ray beam was focused by Kirkpatrick–Baez (KB) optics (JTEC Corporation) using total reflection mirrors located ∼45 m downstream of the slits. The KB mirror design parameters are summarized in Table 1 ▸. The aperture, positioned just in front of the KB mirror, had a size of 270 µm (H) × 315 µm (V) adjusted to illuminate the entire effective area of the mirror. The KB mirrors were housed in an acrylic chamber in a helium gas atmosphere, with 5 µm-thick polyimide windows mounted at the X-ray entrance and exit of the chamber. An ion chamber was placed immediately after the acrylic chamber to monitor incident X-ray intensities. The samples selected for the evaluation of spatial resolution and sensitivity were tantalum (Ta) test charts with thickness values of 200 nm (XRESO-50, NTT Advanced Technology Corp.) and 6 nm (GS20-2, NTT Advanced Technology Corp.) and silica particles (QSG-30, Shin-Etsu Chemical Co. Ltd) with a diameter of approximately 30 nm which were dispersed on a 500 nm-thick SiN membrane. The samples were positioned on piezo stages at the location of a focal point in a vacuum chamber. A 25 µm-thick polyimide window was mounted at the X-ray entrance of the sample chamber. Spatial filters were placed in front of the sample to eliminate parasitic scattering from the focusing mirror (Takahashi *et al.*, 2013 ▸). Silicon slits with a length of 100 µm per side were used as a spatial window. The CITIUS detector used in this study has 840 kpixels and is composed of three sensor modules. Each module has 384 × 728 pixels. The CITIUS detector is mounted on a vacuum flange and connected to the sample chamber through a flight tube, which ensures there is no window between the sample and the detector sensor. It was located ∼2.44 m downstream of the sample. The vacuum was evacuated from the port near the sample to <1 Pa. The diffraction patterns from each sample were collected with a step size of 150 nm and a perfect grid of 17 × 17 scanning points with 1 s exposure per point. For the Ta test charts with a thickness of 200 nm, the slit width was varied from 10 µm to 30 µm in the horizontal direction and from 30 µm to 150 µm in the vertical direction. For the 6 nm-thick Ta test charts and silica particles, the slit width was 30 µm (H) × 150 µm (V).

## 3. Diffraction pattern of 200 nm-thick Ta test chart

**Figure 2:**
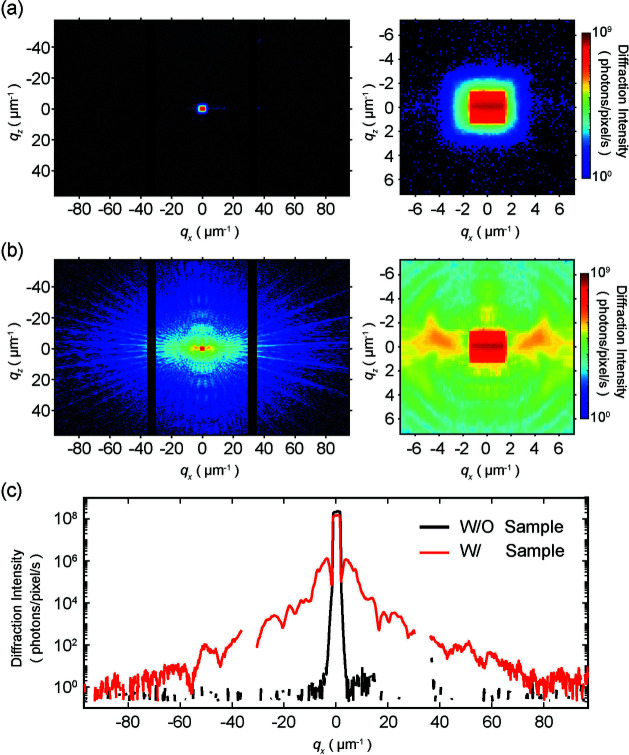
(*a*) Diffraction pattern without the sample, showing overall (left) and an enlarged view (right) of the low-spatial frequency region. (*b*) Diffraction patterns of the 200 nm-thick test chart, showing overall (left) and an enlarged view (right) of the low-spatial frequency region. (*c*) One-dimensional intensity distribution of the diffraction patterns shown in (*a*) and (*b*) along the *q*_*x*_ direction at *q*_*z*_ = 0.

Fig. 2 ▸(*a*) shows a diffraction pattern taken without a sample, where only the intensity distribution of the direct beam can be seen. The intensity around the direct beam was suppressed by a spatial filter. Fig. 2 ▸(*b*) shows one of the ptychographic diffraction patterns from the 200 nm-thick test chart and its low spatial frequency range expansion. The slit width was 30 µm (H) × 150 µm (V), and the flux at the sample position was ∼2.6 × 10^10^ photons s^−1^. The maximum intensity per pixel was ∼250 Mcounts s^−1^. Fig. 2 ▸(*c*) shows the horizontal intensity profile along the *q*_*x*_ direction at *q*_*z*_ = 0 with and without the sample. Diffraction patterns were measured with a high signal-to-noise ratio, and weak diffraction intensity patterns were observed in the high-spatial-frequency range, indicating a one-photon level of sensitivity and handling count rates per pixel.

## 4. Image reconstruction

The diffraction patterns collected for this experiment consist of 1225 pixels in the horizontal direction (*q*_*x*_) and 728 pixels in the vertical direction (*q*_*z*_), which includes the gap between the sensors. To ensure equal pixel dimensions, an image size of 1225 × 1225 pixels was used for the reconstruction, where a value of 0 was assigned for 497 pixels in the high-*q*_*z*_ region. The image reconstruction of the 200 nm-thick Ta test chart was performed using an extended ptychographical iterative engine (ePIE) (Maiden & Rodenburg, 2009 ▸) extended to a mixed-state model (Thibault & Menzel, 2013 ▸) and an algorithm for lateral position correction using the gradient of intensity patterns (Dwivedi *et al.*, 2018 ▸), which is referred to as the IG method in this paper. A function propagating 0.5 mm downstream from a circular aperture of diameter 300 nm was used as the initial probe function, and 700 iterations were performed using three mixed-state probe modes. However, the IG method did not perform well for the image reconstruction of the 6 nm-thick Ta test chart and silica particles due to weak scattering intensity from the sample. To address this, the weak phase object approximation (Dierolf *et al.*, 2010 ▸) and orthogonal probe relaxation (OPR) (Odstrcil *et al.*, 2016 ▸) were used. Although less accurate than the IG method, OPR can correct for irradiation position deviation by treating it as a probe variation. The image reconstruction of the 6 nm-thick Ta test chart and silica particles was performed using ePIE with OPR extended to a mixed-state model (m-s OPR) (Eschen *et al.*, 2022 ▸). To reconstruct the image of the 6 nm-thick Ta test chart and silica particles, the probe function obtained from the reconstruction of the 200 nm-thick Ta test chart with a slit size of 30 µm × 150 µm was used as the initial probe function. Three mixed-state probe modes, each with three eigenprobes, were utilized, and 540 iterations were performed for the 6 nm-thick Ta test chart, while 740 iterations were performed for the silica particles. In all reconstructions, the initial object function had a real part of 1 and an imaginary part of 0, and the pixel size of all reconstructed images was 5.2 nm.

### 4.1. 200 nm-thick Ta test chart

**Figure 3:**
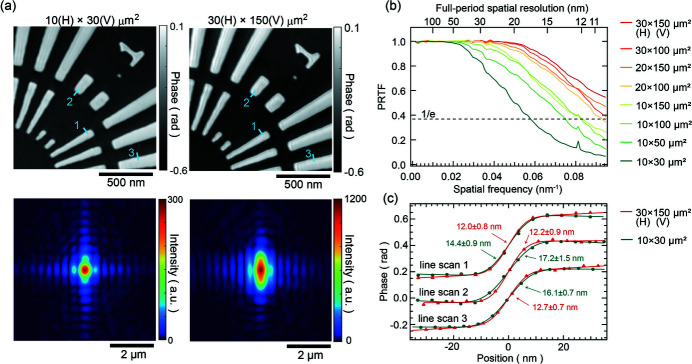
(*a*) Reconstructed phase images (top) and probe intensity images of the first mode in three mixed-state modes (bottom) of a 200 nm test chart at slit widths of 10 µm (H) × 30 µm (V) (left) and 30 µm (H) × 150 µm (V) (right). (*b*) Dependence of the phase retrieval transfer function of the reconstructed image of the 200 nm-thick test chart on slit width. (*c*) Line profiles along the colored lines in the reconstructed images of (*a*). The FWHM values, obtained by fitting with the error function, are also displayed.

Fig. 3 ▸(*a*) depicts the sample phase and probe intensity images of the first mode in three mixed-state modes reconstructed from the diffraction intensity patterns measured with a slit width of 10 µm (H) × 30 µm (V) (left) and 30 µm (H) × 150 µm (V) (right). Both images successfully reconstructed a minimum structure of 50 nm in the sample. The probe intensity distribution obtained with a slit size of 10 µm (H) × 30 µm (V) resembled that of the Fraunhofer diffraction intensity for a rectangular aperture, and it was focused near the diffraction limit. The focal spot size was measured to be 321 nm (H) × 428 nm (V) full width at half-maximum (FWHM). In contrast, when the slit size was increased to 30 µm (H) × 150 µm (V), the vertical direction exhibited a larger geometric reduction size compared with the diffraction-limited focusing size. The measured focal spot size was found to be 343 nm (H) × 900 nm (V) at the FWHM. The spatial resolution was evaluated using the phase retrieval transfer function (PRTF) (Chapman *et al.*, 2006 ▸), averaged over the 17 × 17 scan positions. Since only pixel values containing diffraction data were used, spatial frequency regions higher than ∼0.057 nm^−1^ were valid only for the horizontal direction. The slit-width dependence of the PRTF curve is shown in Fig. 3 ▸(*b*), indicating that spatial resolution improves with increasing slit width. When the slit size was 30 µm (H) × 150 µm (V), the corresponding flux was approximately 2.6 × 10^10^ photons s^−1^, achieving a better full-period spatial resolution than 10.5 nm for the horizontal direction. Furthermore, line profiles for each image were analyzed, as shown in Fig. 3 ▸(*d*) with resolutions of approximately 16 nm and 12 nm for slit sizes of 10 µm (H) × 30 µm (V) and 30 µm (H) × 150 µm (V), respectively.

Table 2 ▸ provides a summary of the flux, beam size at the sample position, and the percentage of each mode in the three mixed-state modes for each slit size. As the slit size expands, the first mode’s percentage decreases while higher-order mode percentages increase. It has been reported that increasing the number of photons in the first mode can improve spatial resolution (Burdet *et al.*, 2016 ▸). This trend is consistent with our findings. As the slit size increases, the step size of the diffraction intensity pattern measurement remains constant, leading to an increased beam overlap ratio. In addition to the heightened flux of the first mode, the increased overlap rate may also enhance the convergence of phase retrieval calculations (Bunk *et al.*, 2008 ▸) and contribute to the improved resolution. The spatial coherence length should be sufficiently coherent in the vertical direction for the 150 µm size. However, the reduced flux of the first modes can be due to vibrations in the monochromator’s first crystal. When the slit size is 30 µm (H) × 150 µm (V), the reconstructed image exhibits line artifacts not observed in the 10 µm (H) × 30 µm (V) configuration, and the slightly poorer phase quantification is likely attributable to the monochromator’s vibration. The exact cause of this phenomenon remains unclear.

### 4.2. 6 nm-thick Ta test chart and silica particles

**Figure 4:**
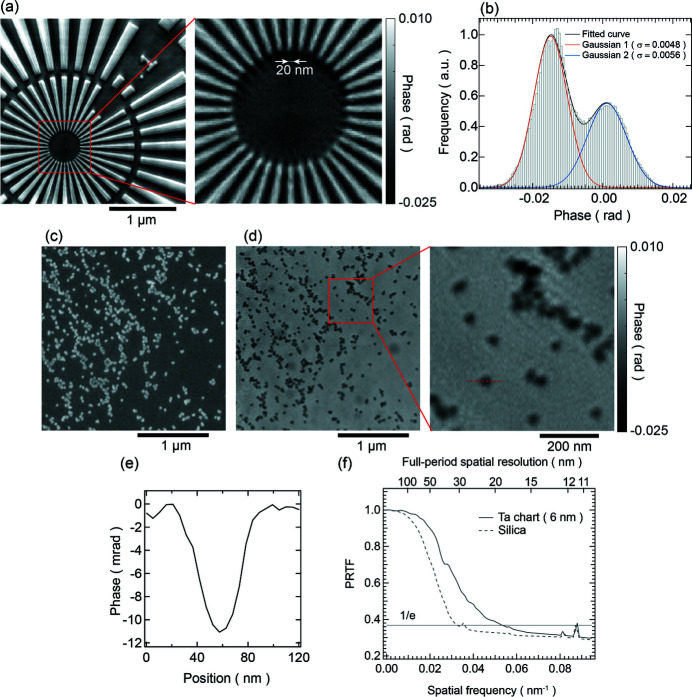
(*a*) Reconstructed phase and magnified image of the 6 nm-thick Ta test chart. (*b*) Histogram of the phase distribution in (*a*), fit with a composite function consisting of two Gaussian functions. (*c*) FE-SEM image of silica particles with an approximate diameter of 30 nm. (*d*) Ptychographic phase and magnified images corresponding to the same field of view as in (*c*). (*e*) Cross-sectional profile of the red dotted line in (*d*). (*f*) Phase retrieval transfer function for the reconstructed images of the 6 nm-thick Ta test chart and silica particles.

Fig. 4 ▸(*a*) displays the reconstructed image of the 6 nm-thick Ta test chart. A minimum structure of 20 nm can be resolved. However, line artifacts, also observed in the 200 nm-thick Ta test chart, are present, with additional artifacts visible in the center of the magnified image. These artifacts are believed to result from the angular oscillation of the monochromator. Fig. 4 ▸(*b*) presents the histogram of the phase distribution corresponding to Fig. 4 ▸(*a*). The histogram was fit using a composite function comprising two Gaussian functions. Phase resolution was determined by measuring the standard deviation (σ) of the Gaussian fit, as defined by Putkunz *et al.* (2014 ▸). Based on this definition, the phase resolution of the current image is superior to 0.006 rad, which, to our knowledge, represents the finest phase resolution achieved by PCDI thus far. Moreover, the interval at the peak top position is 0.016 rad. The theoretical value of the 6 nm-thick Ta phase shift for 6.5 keV X-rays is 0.012 rad. The minor discrepancy of approximately 0.004 rad from the theoretical value is thought to be a reconstruction error caused by the m-s OPR method. Figs. 4 ▸(*c*) and 4 ▸(*d*) show the field-emission scanning electron microscopy (FE-SEM) and reconstructed phase images of the silica particles with a diameter of ∼30 nm, respectively, indicating that the sample image is reconstructed at the same position as the FE-SEM image. Fig. 4 ▸(*e*) shows the cross-sectional profile of the particle in Fig. 4 ▸(*d*). The phase shift of the 30 nm silica particles for 6.5 keV X-rays was estimated to be 0.0089 rad, indicating that a similar phase shift was reconstructed. The present results are comparable with the best sensitivity reported to date (Lima *et al.*, 2013 ▸) and are of a high standard for spatial resolution. Fig. 4 ▸(*f*) presents the PRTF curves for the reconstructed images of the 6 nm-thick test chart and the silica particles. Based on the 1/e criterion, the respective resolutions are determined to be 18.7 nm and 29.9 nm.

## 5. Conclusion

In this study, PCDI measurements were performed using the high-speed X-ray imaging detector CITIUS at SPring-8 BL29XUL, in which 6.5 keV X-rays were focused by total reflection focusing mirrors, and a flux of ∼2.6 × 10^10^ photons s^−1^ was obtained at the sample plane. Diffraction intensity data were collected at up to ∼250 Mcounts s^−1^ pixel^−1^ without saturation of the detector. With a spatial resolution of >10.5 nm, 200 nm-thick Ta test chart phase images were reconstructed. Additionally, the phase images of the 6 nm-thick Ta test chart with a minimum size of 20 nm and silica particles with a diameter of ∼30 nm have been reconstructed, which are extremely weak phase objects with a phase shift of ∼0.01 rad. The present results show a high standard of reconstruction with high spatial resolution and high sensitivity. The CITIUS detector will be an indispensable imaging device for sample observation in various fields using low-emittance synchrotron radiation sources.

## Figures and Tables

**Table 1:** Parameter values for the KB mirrors

	Vertical focusing mirror	Horizontal focusing mirror
Glancing angle (mrad)	3.5	3.0
Mirror length (mm)	90	90
Focal length (m)	0.595	0.490

**Table 2:** Flux, beam size at the sample position, and percentage of each mode in the three mixed-state modes for varying slit sizes

		Probe size of first mode (nm)		Percentage of mixed-state mode		
Slit size (H × V) (µm)	Flux (photons s^−1^)	H	V	1st	2nd	3rd
30 × 150	2.6 × 10^10^	343	900	69.7	18.4	11.9
30 × 100	1.7 × 10^10^	328	582	68.4	19.9	11.7
20 × 150	1.6 × 10^10^	337	928	69.0	22.9	8.1
20 × 100	1.0 × 10^10^	320	652	82.2	10.4	7.4
10 × 150	5.2 × 10^10^	330	958	74.1	20.0	5.9
10 × 100	3.4 × 10^9^	317	660	75.3	21.1	3.6
10 × 50	1.5 × 10^9^	322	449	76.4	18.4	5.2
10 × 30	6.5 × 10^8^	321	428	86.3	8.8	4.9
